# Relationships between Selected Physiological Factors and Milking Parameters for Cows Using a Milking Robot

**DOI:** 10.3390/ani10112063

**Published:** 2020-11-07

**Authors:** Marian Kuczaj, Anna Mucha, Alicja Kowalczyk, Ryszard Mordak, Ewa Czerniawska-Piątkowska

**Affiliations:** 1 Faculty of Biology and Animal Science, Wrocław University of Environmental and Life Sciences, ul. J. Chełmońskiego 38C, 51-630 Wrocław, Poland; marian.kuczaj@upwr.edu.pl; 2 Department of Genetics, Wroclaw University of Environmental and Life Sciences, Kożuchowska 7, 51-631 Wroclaw, Poland; anna.mucha@upwr.edu.pl; 3 Department of Environment, Animal Hygiene and Welfare, Wrocław University of Environmental and Life Sciences, J. Chełmońskiego 38C, 51-630 Wrocław, Poland; 4 Department of Internal Diseases, Faculty of Veterinary Medicine, Wrocław University of Environmental and Life Sciences, Pl. Grunwaldzki 47, 50-366 Wrocław, Poland; ryszard.mordak@upwr.edu.pl; 5 Department of Ruminant Sciences, Faculty of Biotechnology and Animal Breeding, West Pomeranian University of Technology in Szczecin, Klemensa Janickiego 29, 71-270 Szczecin, Poland; ewa.czerniawska-piatkowska@zut.edu.pl

**Keywords:** cattle, milking robot, milking parameters, milk yield and composition, number and stage of lactations, time of day and year

## Abstract

**Simple Summary:**

Modern milking robots create the possibility for better organization of work. In recent years, in selection indexes constructed for Holstein Friesian cattle, milking capacity features have become of great importance. This study showed a negative correlation between the milk yield and the content of its components. The quantity of milk released from the hind quarters was found to be higher than that determined for the fore quarters. At the same time, the milk flow rate turned out to be statistically significantly higher in the front quarters as compared to the back quarters. The speed of milking is an individual feature; therefore, it is important that the milking machines have the ability to control the negative pressure during the milking of individual quarters, and thus react to the rate of milk flow from individual teats.

**Abstract:**

The aim of the study was to determine the effect of the number and stage of lactations, time of day and calving season of cows on milk yield from a single milking, average milking time, average milking per minute, daily milking frequency and the relationship between the tested parameters of quarter milking. The study included a herd of 65 Polish Holstein Friesian black and white cows used in a free-range barn located in south-west Poland. The animals were kept in proper welfare conditions, fed using the partly mixed ration (PMR) method on the feeding table. The milk was obtained using the Lely-Astronaut A4 Automatic Milking System (AMS). The animals on the dairy cattle farm were used in the range from the first to the seventh lactation, i.e., at the age of 2.0 to approximately 10 years. In this study, the amount of milk yielded from the hind quarters was statistically significantly higher (*p* < 0.05) than the trait determined for the front quarters. At the same time, the milk flow rate was statistically significantly higher (*p* < 0.05) in the front quarters compared to the rear quarters. The daily milk yield in right rear (RR) and left rear (LR) hind quarters was higher by 1.0 kg of milk, respectively, than in right front (RF) and left front (LF) fore quarters. The milking time of the RR and LR hind quarters during the day was longer by 104.9 and 128.8 s, respectively, than the RF and LF fore quarters. The milking speed of the RR and LR hind quarters during the day was lower by 0.2 and 1.12 g/s, respectively, than in the RF and LF fore quarters. The values of the correlation between the yields of milk and its components obtained in this study were high and positive. Correlations between the milk yield and the content of its components were negative. The obtained results confirmed that the natural physiological variability of the udder and teats structure, as well as the course of lactation, significantly affects the individual composition and milk flow during milking. The ability to regulate the milk flow by adjusting the appropriate negative pressure during the robot’s operation, in the observed variability of individual lobes of the mammary gland, increases the efficiency of milking and, as a result, reduces the risk of mastitis in cows.

## 1. Introduction

The main reason for starting the development of automatic milking in the 1980s was the need for improved labor efficacy due to the growing costs of labor in many dairy countries [1,2]. Much of the significant advancement in the twentieth-century dairy industry has focused on maximizing milk production. Automatic milking systems (AMSs) and automatic milking rotary (AMR) parlors represent the most recent technological efforts, offering the potential for frequent milking events without depending on human labor [3], and the adoption of milking robots is spreading rapidly. One of the benefits of automatic milking systems (AMSs) is increased milk yield from more frequent milking. An increase from 6 to 25% in complete lactations has been shown when milking frequency increases from two times to three times per day [4].

To date, scientific research has examined various aspects of AMS technology and its effect on milk quality, herd health, welfare, behavior and management. Multiple differences exist between AMS and conventional parlors, making targeted research on new milking systems necessary. The fully automatic milking process of the AMS, which milks the udder on a quarter basis, and the automatic teat-cleaning and milking cup-attachment processes have the potential to affect milk variables and udder health. Cows must be motivated to voluntarily approach and enter AMS milking stalls, as they are no longer brought to the milking parlor two or three times daily by human handlers.

Despite the growing popularity of AMSs, little research has focused on milking parameters or their relationship with physiological factors. So far, much attention has been paid to the economic performance of introducing this technology in dairy production [5,6].

The aim of the study was to determine the effect of the number and stage of lactations, time of day and calving season of cows on milk yield from a single milking, average milking time, average milking per minute, daily milking frequency and the relationship between the tested parameters of quarter milking.

## 2. Material and Methods

In accordance with applicable law, the conducted research did not require the approval of the local ethical committee. All procedures included in this experiment were routine activities in the herd.

The study included a herd of 65 Polish Holstein Friesian black and white cows used in a free-range barn located in south-west Poland. The animals were kept in proper welfare conditions and fed using the partly mixed ration (PMR) method on the feeding table. Depending on the daily milk yield, the cows additionally took the concentrated mixture individually in the milking box.

The cows were fed in two ways:

1. The basic mixture for the whole herd was fed from the feed table as standardized PMR (partly mixed ration) calculated on the average production of about 25 kg of milk per cow and was based on homemade feeds such as high-quality corn silage, alfalfa grass silage, moisture corn, sugar beet pulp, alfalfa grass hay, barley straw, rapeseed meal, soybean meal, minerals and vitamins. The entire dose contained approximately 45% dry matter. PMR was supplemented with a commercial mix of concentrates for lactating animals according to their daily milk yield.
2. The compound feed was given to cows during milking on a robotic milking station. The amount of this feed depended on the current daily milk yield. The concentrate feed at the feed stand has been developed with the company’s aromatic and flavor additive.

The cows had free access to fresh water.

The milk was obtained using the Lely-Astronaut A4 Automatic Milking System (AMS). The animals on the dairy cattle farm were used in the range from the first to the seventh lactation, i.e., at the age of 2.0 to approximately 10 years.

Test data were obtained from the Lely T4C management system (recorded by the AMS) and the SYMLEK recording system. Basic descriptive statistics on milk yield, milking time, milking speed and consumption of concentrates during milking (after the rejection of visits to the robot ended with refusal) were determined based on 40 705 single milkings of 82 cows milked between November 2015 and December 2018 (Table 1).

The research took into account the following features: daily milking frequency—number of milking cows per day, total and quarter milking time expressed in seconds (s), total and quarter milk yield during milking from one cow (in kg), fat and protein content in milk from a single milking from one cow (expressed in percent), average milk yield per minute obtained from the milking of one cow (kg/min) and consumption of concentrate in the box for a single milking, expressed in grams.

The following markings have been introduced for individual quarters of the list: the left front (LF), left rear (LR), right front (RF) and right rear (RR) teat.

The individual parameters were analyzed depending on the following factors:

(1) Number of milkings in subsequent lactations: 1 (n = 15,599), 2 (n = 11,271), 3 (n = 7731), 4 (n = 33,883), ≥5 (n = 2221);
(2) Lactation stage: ≤100 days (n = 20 054), 101–200 days (n = 13,571), 201–305 days (n = 7080);
(3) Time of the day of milking: 00:01–08:00 (n = 13,770), 08:01–16:00 (n = 13,277), 16:01–00:00 (n = 13,658);
(4) Calving season: spring–summer—from March to August (n = 26,544), fall–winter—from September to February (n = 14,161).

### Statistical Analysis

The statistical analysis was performed using R software version 3.4.4 (R Core Team, Vienna, Austria) [7]. The basic descriptive statistics of considered traits (milk yield, milking duration, milking speed, concentrates intake, fat yield, protein yield, milking frequency) were determined with the *pastecs* package [8]. The compliance of the distribution of analyzed traits with the normal distribution was verified using the Cramer von Mises test implemented in the *nortest* package [9]. The statistical significance of differences in milk yield, milking duration and milking speed between particular teats was verified with the Kruskal–Wallis non-parametric analysis of variance in the *agricolae* package [10].

Moreover, Pearson’s correlation coefficients for the considered features for single milkings and for the daily milking were calculated.

## 3. Results

The basic descriptive statistics of milk yield, milking duration, milking speed and concentrates intake were determined based on 40 705 single milkings of 82 cows (Table 1). Table 2 shows the basic descriptive statistics of milk yield, milk duration and milking speed for each teat. The milk yield ranged from 3.30 to 29.20 kg for single milkings, from 0.5 to 7 kg for the left front, left rear and right rear teats and from 0.5 to 6.9 kg for the right front teat. The average milk yield of all single milkings stood at 10.37 kg with a standard deviation equal to 2.56 kg. A statistically significant influence of the position of the teat on the milk yield was observed (Kruskal–Wallis test, *p*-value < 2.2 × 10^−16^). The post hoc analysis showed significant differences in milk yield for front teats compared to rear ones. The milk yield was characterized by the lowest variability among the analyzed characteristics with the variation coefficient at the level of 24.71% for single milkings.

The milking duration changed in the range from 101 to 819 s for single milkings, from 100 to 698 s for the left front teat, from 51 to 819 s for the left rear teat, from 100 to 603 s for the right front teat and from 100 to 659 s for the right rear teat. The average milking duration of all single milkings was at the level of 237.92 s with a standard deviation equal to 84.12. A statistically significant influence of the position of the teat on the milking duration was found (Kruskal–Wallis test, *p*-value < 2.2 × 10^−16^). The post hoc analysis showed significant differences in milk duration for front teats compared to rear ones.

The milking speed ranged from 9.62 to 182.5 g/s for single milkings, from 1.9 to 46.3 g/s for the left front teat, from 1.6 to 49.6 g/s for the left rear teat, from 1.8 to 42.3 g/s for the right front teat and from 2 to 49.6 g/s for the right rear teat. The average milking speed of all single milkings stood at 47.66 grams per second with a standard deviation equal to 16.89. A statistically significant influence of the position of the teat on the milking speed was observed (Kruskal–Wallis test, *p*-value < 2.2 × 10^−16^) for all four positions. The milking speed was characterized by the highest variability among the analyzed characteristics with the variation coefficient at the level of 35.44% for single milkings.

The concentrates intake reached a mean value equal to 1243.49 grams with a standard deviation of 346.35 for the single milking.

The basic descriptive statistics of milk yield, milking duration, milking speed, concentrates intake, fat yield, protein yield and milking frequency were determined based on 14 618 daily milkings of 82 cows (Table 3 and Table 4).

The average milk yield of all daily milkings stood at 28.88 kg with a standard deviation equal to 12.86 kg. The daily milk yield ranged from 3.4 to 79.7 kg. The daily fat and protein yield ranged from 2.6 to 5.3 kg and from 2.7 to 4.2 kg with the average yield equal to 4.18 and 3.45 kg, respectively. The daily duration of milking lasted from 105 to 2502 s with an average of 662.49 s. The daily frequency of milking was from 1 to 6 with an average of 2.78.

The differences between daily milk yield, milk duration and milking speed for left front, right front, left rear and right rear teats were analogical to single milkings.

The dataset of single milkings was divided according to lactation number and stage, calving season and time of day. Then, the influence of the above factors on the considered traits was verified.

Table 5 presents the mean values and standard deviations of the analyzed traits depending on the lactation number. Lactation numbers 5, 6 and 7 were treated as one set. Statistical significance of the impact of the lactation number on all analyzed traits was found.

The highest milk yield (11.73 kg) was observed for the fourth lactation and the lowest (9.41 kg) for the first lactation. The average milk yield in a single milking was comparable for the second (10.67 kg) and fifth, sixth and seventh lactation (10.77 kg).

The duration of milking varied significantly depending on the lactation number. A single milking lasted the longest in the fifth, sixth and seventh lactation (280.78 s), and then shortened from the first (251.09 s) to the fourth lactation (197.52 s).

The lowest milking speed was noted for the first (40.94 g/s) and for the last lactations (40.61 g/s). The milking speed increased statistically significantly from the second (49.86 g/s) to the fourth lactation (61.78 g/s).

The concentrates intake increased from 1189.43 for the first lactation to 1353.88 g for the fourth lactation, and then fell to 1306.44 g for the fifth, sixth and seventh lactation. All the differences were statistically significant.

The mean values and standard deviations of the analyzed traits depending on the lactation stage, calving season and time of the day are shown in Table 6.

A statistically significant decrease in milk yield and milking duration was noted with each subsequent lactation stage. A similar tendency was shown for the milking speed, but differences in mean values were not statistically significant. The concentrate intake was the highest in the second stage of lactation and the smallest in the first stage. The averages of this trait differed statistically significantly for all three stages of lactation.

The mean values of the considered traits were higher in the spring–summer calving season than in the autumn–winter season, but for the milking duration, the observed difference was not statistically significant.

Milk yield and milking duration dropped statistically significantly in subsequent parts of the day. The milking speed between 16:01 and 0:00 was significantly lower than between 0:01 and 8:00 and 8:01 and 16:00. There were no statistically significant differences in the average concentrate intake at particular times of the day.

Table 7 presents the mean values and standard deviations of the analyzed traits for daily milkings depending on the lactation number. Lactation numbers 5, 6 and 7 were treated as one set. Statistical significance of the impact of the lactation number on all analyzed traits was found.

The highest milk yield (32.86 kg) was observed for the third lactation and the lowest (27.29 kg) for the first lactation. The highest fat and protein yields were shown for the fifth, sixth and seventh lactation (4.45 kg and 3.54 kg, respectively).

The daily duration of milking lasted the longest in the fifth, sixth and seventh lactation (731.95 s), and the shortest in the fourth lactation (504.26 s).

The lowest milking speed was noted for the first (43.46 g/s) and for the last lactations (44.14 g/s). The milking speed increased statistically significantly from the second (53.23 g/s) to the fourth lactation (63.17 g/s).

The concentrates intake for the third lactation (3669.26 g) was statistically significantly higher than for all other lactation groups.

The highest milking frequency was found for the first (2.97 milkings per day) and for the third lactation (2.90 milkings per day). Milking frequency was statistically significantly lower for all other lactations and ranged from 2.55 for the fourth lactation to 2.64 milkings per day for the second lactation.

The mean values and standard deviations of the analyzed traits for daily milkings depending on the lactation stage, calving season and time of the day are shown in Table 8.

A statistically significant decrease in milk yield and milking duration was noted with each subsequent lactation stage. A similar tendency was shown for the milking speed, but differences in mean values for the first and the second lactation stage were not statistically significant. The fat and protein yields increased statistically significantly with the lactation stage. The concentrate intake yield and the milking frequency decreased statistically significantly with the lactation stage.

The milk and protein yield, milking speed and concentrates intake were statistically significantly higher in the spring–summer calving season than in the autumn–winter season. The fat yield was statistically significantly lower in the spring–summer calving season than in the autumn–winter season. The differences observed for the milking duration and milking frequency were not statistically significant.

The correlation matrix of all considered traits for single milkings is shown in Table 9. All determined correlation coefficients were statistically significant. The results obtained were in line with the obvious expectations. The milking duration was strongly positively correlated with left rear and right rear teat milking duration (0.89 and 0.82, respectively) and moderately correlated with left front and right front teat milking duration (0.67). The milking speed was moderately negatively correlated with milking duration (−0.68). A strong positive correlation was found for the milk yield and left front, left rear, right front and right rear teat milk yield. Analogous relationships were demonstrated for daily milkings, shown in Table 10. Furthermore, the concentrates intake was strongly positively correlated with all milk yield and milking duration traits.

## 4. Discussion

Daily milk yield in a given lactation period depends on the nutritional status of the cow and the level of nutrition that will cover the animal’s maintenance and production needs [2], but also on the frequency of milking [11].

The speed of milking is an individual feature; therefore, it is important that the milking machines have the ability to control the negative pressure during the milking of individual quarters, and thus react to the rate of milk flow from individual teats. Rodenburg [12] states that the improvement of the efficiency of milking robots can be achieved indirectly by selecting animals that have correctly positioned teats, as well as milking evenly and quickly. Research by Sewalem et al. [13] suggests that individual differences in milking speed are small. This research brings a different conclusion. The response to changes in the milk flow rate may affect the condition of the udder tissues and, indirectly, also the efficiency of cows [14]. Moreover, the frequency of milking has a strong influence on udder health and the production level of the herd. Bruckmaier and Hilger [11] analyzed the length of intervals between individual milkings on the yield and speed of milk given. They showed that short intervals had a negative effect on the health of the udder, but too long an interval caused a reduction in daily and lactation efficiencies. The use of individual milking intervals is possible in the case of automated milking systems. At the same time, Szymik et al. [15] emphasize that the Holstein Friesian breed seems to milk faster than the Simental and Jersey. Sewalem et al. [13] suggest that a slower milking rate may be correlated with the animal’s temperament. Only cows of the Polish Holstein Friesian breed participated in this study; however, large individual differences were found in terms of milking speed. The values of daily yield obtained in this study, the rate of milk flow and its quantity from individual quarters are consistent with the results obtained in the works of Bruckmaier and Hilger [11], Vetharaniam et al. [16] and Wieland et al. [14].

On farms using milking robots, moderate-temperament, fast-milking, calm and cooperative cows are also desired. Such cows will move efficiently in the robot, eagerly take the fodder after giving up the milk and then leave the machine, allowing the next cows to milk [17].

In studies by Weiss et al. [18], milk yield and maximum milk flow rate were higher in the hind quarters compared to the front quarters. In this study, the amount of milk yielded from the hind quarters was statistically significantly higher than the trait determined for the front quarters. At the same time, the milk flow rate was statistically significantly higher in the front quarters compared to the rear quarters.

In some cases, the quality of the milk may decrease by observing the cows less often for their welfare and health, which may deteriorate in this situation. Automatic milking robots require constant access to a qualified service [19,20]. One should also remember technological proficiency and the appropriate habituation of animals to the milking equipment. Modern milking robots create the possibility for better organization of work. In recent years, in selection indexes constructed for Holstein Friesian cattle, milking capacity features have become of great importance.

The values of the correlation between the yields of milk and its components obtained in this study were high and positive. Correlations between the milk yield and the content of its components, as expected, were negative. Similar results were also obtained by Weiss et al. [18], Edwards et al. [21] and Costa et al. [22].

## 5. Conclusions

Modern milking robots create the possibility for better organization of work. In recent years, in selection indexes constructed for Holstein Friesian cattle, milking capacity features have become of great importance. The AMS provides dairy farmers with a wide database on the yield and chemical composition of milk as well as milking parameters from individual cows in the herd, which, after analysis, allows for more effective management of the milk yield of cows and the health of the mammary gland. This study showed a negative correlation between the milk yield and the content of its components. The quantity of milk released from the hind quarters was found to be higher than that determined for the fore quarters. At the same time, the milk flow rate was statistically significantly higher in the front quarters as compared to the back quarters. The speed of milking is an individual feature; therefore, it is important that the milking machines have the ability to control the negative pressure during the milking of individual quarters, and thus react to the rate of milk flow from individual teats. The results, statistical data and observations disclosed in our study, also in relation to the research of other authors, indicate that they may be useful for further research on the impact of using milking robots on the health of the mammary gland, occurrence of mastitis and the length of use of milking cows.

## Figures and Tables

**Table 1:** Basic descriptive statistics of considered traits for single milkings.

	Trait			
	Milk Yield [kg]	Milking Duration [s]	Milking Speed [g/s]	Concentrates Intake [g]
**n**	40 705	40 705	40 705	40 705
**Mean**	10.37	237.92	47.66	1243.49
**Median**	9.90	220.00	45.40	1188.00
**Minimum**	3.30	101.00	9.62	104.00
**Maximum**	29.20	819.00	182.50	2889.00
**SD**	2.56	84.12	16.89	346.35
**VC [%]**	24.71	35.36	35.44	28.85

n = number of milkings, SD = standard deviation, VC = variation coefficient (presented as a percentage).

**Table 2:** Basic descriptive statistics of considered traits for single milkings from the left front (LF), left rear (LR), right front (RF) and right rear (RR) teats.

	Trait											
	Milk Yield [kg]				Milking Duration [s]				Milking Speed [g/s]			
	LF	LR	RF	RR	LF	LR	RF	RR	LF	LR	RF	RR
**n**	40 705				40 705				40 705			
**Mean**	2.4 ^a^	2.7 ^b^	2.4 ^a^	2.7 ^b^	167.7 ^c^	213.9 ^a^	167.5 ^b^	205.2 ^a^	15.3 ^a^	14.0 ^b^	14.9 ^c^	14.3 ^d^
**Median**	2.3	2.6	2.3	2.6	151.0	195.0	154.0	194.0	15.1	13.7	14.6	13.5
**Minimum**	0.5	0.5	0.5	0.5	100.0	51.0	100.0	100.0	1.9	1.6	1.8	2.0
**Maximum**	7.0	7.0	6.9	7.0	698.0	819.0	603.0	659.0	43.6	49.6	42.3	49.6
**SD**	0.8	1.0	0.8	1.0	64.2	84.7	54.9	67.4	5.6	5.8	5.2	5.8
**VC [%]**	33.7	36.8	32.1	35.3	38.3	39.6	32.8	32.9	36.8	41.3	35.0	40.3

Mean values differing statistically significantly between teats were marked by different letters a,b,c,d (*p*-value < 0.05). n = number of milkings, SD = standard deviation, VC = variation coefficient (presented as a percentage).

**Table 3:** Basic descriptive statistics of considered traits for daily milkings.

	Trait						
	Milk Yield [kg]	Fat Yield [kg]	Protein Yield [kg]	Milking Duration [s]	Milking Speed [g/s]	Concentrates Intake [g]	Milking Frequency [milkings/day]
**n**	14 618	14 618	14 618	14 618	14 618	14 618	14 618
**Mean**	28.88	4.18	3.45	662.49	50.47	3462.59	2.78
**Median**	29.00	4.13	3.46	606.00	47.98	3588.00	3.00
**Minimum**	3.40	2.60	2.70	105.00	9.62	104.00	1.00
**Maximum**	79.70	5.30	4.20	2502.00	182.50	9261.00	6.00
**SD**	12.86	0.51	0.26	402.27	17.42	1536.34	1.26
**VC [%]**	44.52	12.30	7.65	60.72	34.51	44.37	45.28

n = number of daily milkings, SD = standard deviation, VC = variation coefficient (presented as a percentage).

**Table 4:** Basic descriptive statistics of considered traits for daily milkings from the left front (LF), left rear (LR), right front (RF) and right rear (RR) teats.

	Trait											
	Milk Yield [kg]				Milking Duration [s]				Milking Speed [g/s]			
	LF	LR	RF	RR	LF	LR	RF	RR	LF	LR	RF	RR
**n**	14 618				14 618				14 618			
**Mean**	6.6 ^a^	7.6 ^b^	6.6 ^a^	7.6 ^b^	466.9 ^a^	595.7 ^b^	466.5 ^a^	571.4 ^b^	16.2 ^a^	15.08 ^c^	15.6 ^b^	15.4 ^c^
**Median**	6.5	7.3	6.4	7.4	417.0	525.0	426.5	525.0	16.1	14.50	15.3	14.4
**Minimum**	0.5	0.5	0.5	0.6	100.0	51.0	100.0	100.0	2.2	1.94	2.3	2.9
**Maximum**	19.7	26.1	21.0	22.8	1986.0	2463.0	1644.0	2149.0	40.0	48.15	39.6	44.6
**SD**	3.3	3.8	3.3	3.7	289.0	389.1	272.9	336.3	5.7	5.93	5.3	6.0
**VC [%]**	49.2	50.0	50.0	48.2	61.9	65.3	58.5	58.9	35.2	39.33	33.8	38.9

Mean values differing statistically significantly between teats were marked by different letters a,b,c (*p*-value < 0.05). n = number of milkings, SD = standard deviation, VC = variation coefficient (presented as a percentage).

**Table 5:** Means and standard deviations (SD) of considered traits for single milking in the groups designated by lactation number.

	Trait			
	Milk Yield [kg]	Milking Duration [s]	Milking Speed [g/s]	Concentrates Intake [g]
**1st lactation (n = 15 599)**				
**mean ± SD**	9.41 ^a^ ± 2.33	251.09 ^a^ ± 91.13	40.94 ^a^ ± 13.86	1189.43 ^a^ ± 327.80
**2nd lactation (n = 11 271)**				
**mean ± SD**	10.67 ^b^ ± 2.21	235.44 ^c^ ± 83.86	49.86 ^b^ ± 17.28	1273.71 ^b^ ± 340.71
**3rd lactation (n = 7731)**				
**mean ± SD**	11.06 ^c^ ± 2.70	222.91 ^d^ ± 70.07	52.96 ^c^ ± 16.44	1234.95 ^c^ ± 334.41
**4th lactation (n = 3883)**				
**mean ± SD**	11.73 ^d^ ± 2.87	197.52 ^e^ ± 57.56	61.78 ^d^ ± 15.64	1353.88 ^d^ ± 397.51
**5th, 6th, 7th lactation (n = 2221)**				
**mean ± SD**	10.77 ^b^ ± 2.41	280.78 ^b^ ± 78.04	40.61 ^a^ ± 12.54	1306.44 ^e^ ± 372.55

Mean values differing statistically significantly between teats were marked by different letters a,b,c,d,e (*p*-value < 0.05). n = number of milkings.

**Table 6:** Means and standard deviations of the considered traits for single milkings in the groups designated by lactation stage, calving season and time of day.

	Trait			
	Milk Yield [kg]	Milking Duration [s]	Milking Speed [g/s]	Concentrates Intake [g]
**1st lactation stage (n = 20 054)**				
**mean ± SD**	10.67 ^a^ ± 2.62	245.25 ^a^ ± 86.01	48.03 ± 18.01	1210.61 ^a^ ± 345.99
**2nd lactation stage (n = 13 571)**				
**mean ± SD**	10.27 ^b^ ± 2.54	235.73 ^b^ ± 86.09	47.62 ± 16.46	1292.82 ^b^ ± 344.56
**3rd lactation stage (n = 7080)**				
**mean ± SD**	9.71 ^c^ ± 2.30	221.32 ^c^ ± 71.36	46.69 ± 14.16	1242.05 ^c^ ± 339.62
**spring–summer season (n = 26 544)**				
**mean ± SD**	10.74 ^a^ ± 2.65	239.12 ± 86.98	49.23 ^a^ ± 17.28	1260.36 ^a^ ± 362.56
**autumn–winter season (n = 14 161)**				
**mean ± SD**	9.67 ^b^ ± 2.22	235.66 ± 78.45	44.71 ^b^ ± 15.72	1211.86 ^b^ ± 311.28
**0:01–8:00 (n = 13 770)**				
**mean ± SD**	10.57 ^a^ ± 2.53	241.28 ^a^ ± 85.85	48.04 ^a^ ± 17.00	1241.66 ± 342.99
**8:01–16:00 (n = 13 277)**				
**mean ± SD**	10.52 ^b^ ± 2.68	238.34 ^b^ ± 83.45	48.11 ^a^ ± 17.07	1274.62 ± 357.04
**16:01–0:00 (n = 13 658)**				
**mean ± SD**	10.02 ^c^ ± 2.44	234.11 ^c^ ± 82.86	46.84 ^b^ ± 16.57	1215.06 ± 336.52

Mean values differing statistically significantly between teats were marked by different letters a,b,c (*p*-value < 0.05). n = number of milkings.

**Table 7:** Means and standard deviations (SD) of the considered traits for daily milkings in the groups designated by lactation number.

	Trait						
	Milk Yield [kg]	Fat Yield [kg]	Protein Yield [kg]	Milking Duration [s]	Milking Speed [g/s]	Concentrates Intake [g]	Milking Frequency [milkings/day]
1st lactation (n = 5380)							
mean	27.29 ^a^	4.23 ^a^	3.49 ^b^	728.03 ^a^	43.46 ^a^	3448.70 ^a^	2.90 ^a^
± SD	±11.13	±0.51	±0.25	±424.16	±14.64	±1501.42	±1.28
2nd lactation (n = 4263)							
mean	28.22 ^b^	4.11 ^b^	3.43 ^c^	622.48 ^b^	53.23 ^b^	3367.57 ^a^	2.64 ^b^
± SD	±12.62	±0.52	±0.28	±406.89	±17.75	±1497.84	±1.21
3rd lactation (n = 2602)							
mean	32.86 ^c^	4.02 ^c^	3.42 ^c^	662.32 ^c^	55.11 ^c^	3669.26 ^b^	2.97 ^a^
± SD	±14.65	±0.42	±0.24	±365.02	±16.76	±1538.60	±1.31
4th lactation (n = 1521)							
mean	29.94 ^d^	4.29 ^d^	3.41 ^d^	504.26 ^d^	63.17 ^d^	3456.36 ^a^	2.55 ^b^
± SD	±14.29	±0.54	±0.31	±277.23	±16.12	±1630.63	±1.19
5th, 6th, 7th lactation (n = 852)							
mean	28.08 ^a b^	4.45 ^e^	3.54 ^a^	731.95 ^a^	44.14 ^a^	3405.64 ^a^	2.61 ^b^
± SD	±12.95	±0.50	±0.23	±428.81	±14.55	±1700.58	±1.20

Mean values differing statistically significantly between teats were marked by different letters a,b,c,d,e (*p*-value < 0.05). n = number of daily milkings.

**Table 8:** Means and standard deviations (SD) of the considered traits for daily milkings in the groups designated by lactation stage, calving season and time of day.

	Trait						
	Milk Yield [kg]	Fat Yield [kg]	Protein Yield [kg]	Milking Duration [s]	Milking Speed [g/s]	Concentrates Intake [g]	Milking Frequency [milkings/day]
1st lactation stage (n = 6618)							
mean	32.33 ^a^	4.07 ^a^	3.41 ^a^	743.17 ^a^	51.14 ^a^	3668.41 ^a^	3.03 ^a^
± SD	±13.52	±0.49	±0.25	±436.08	±18.72	±1531.31	±1.29
2nd lactation stage (n = 5005)							
mean	27.85 ^b^	4.19 ^b^	3.45 ^b^	639.18 ^b^	50.93 ^a^	3505.46 ^b^	2.71 ^b^
± SD	±11.95	±0.48	±0.26	±389.71	±17.23	±1544.50	±1.26
3rd lactation stage (n = 2995)							
mean	22.96 ^c^	4.41 ^c^	3.55 ^c^	523.18 ^c^	48.24 ^b^	2936.12 ^c^	2.36 ^c^
± SD	±10.06	±0.54	±0.28	±284.96	±14.30	± 1407.12	±1.07
spring–summer season (n = 9549)							
mean	29.86 ^a^	4.12 ^a^	3.47 ^a^	664.7	51.76 ^a^	3503.50 ^a^	2.78
± SD	±12.98	±0.48	±0.25	±397.04	±17.68	±1474.58	±1.23
autumn–winter season (n = 5069)							
mean	27.02 ^b^	4.28 ^b^	3.41 ^b^	658.34	48.04 ^b^	3385.51 ^b^	2.79
± SD	±12.42	±0.56	±0.28	±411.96	±16.64	±1643.78	±1.31

Mean values differing statistically significantly between teats were marked by different letters a,b,c (*p*-value < 0.05). n = number of daily milkings.

**Table 9:** Pearson’s correlation coefficients of considered traits for single milkings.

	MD	MY	LF MY	LR MY	RF MY	RR MY	LF MD	LR MD	RF MD	RR MD	CI	MS
**MD**	1	0.24	0.14	0.17	0.12	0.15	0.67	0.89	0.67	0.82	0.23	−0.68
**MY**		1	0.79	0.78	0.77	0.81	0.21	0.21	0.24	0.26	0.62	0.46
**LF MY**			1	0.47	0.70	0.53	0.21	0.08	0.14	0.15	0.45	0.42
**LR MY**				1	0.50	0.68	0.10	0.23	0.16	0.13	0.50	0.33
**RF MY**					1	0.49	0.17	0.09	0.29	0.13	0.44	0.41
**RR MY**						1	0.03	0.11	0.10	0.21	0.53	0.40
**LF MD**							1	0.52	0.58	0.54	0.20	−0.42
**LR MD**								1	0.58	0.71	0.19	−0.62
**RF MD**									1	0.62	0.22	−0.44
**RR MD**										1	0.25	−0.56
**CI**											1	0.21
**MS**												1

All correlation coefficients were statistically significant. MD = milking duration, MY = milk yield, LF = left front teat, LR = left rear teat, RF = right front teat, RR = right rear teat, CI = concentrates intake, MS = milking speed.

**Table 10:** Pearson’s correlation coefficients of considered traits for daily milkings.

	MF	MY	LF MY	LR MY	RF MY	RR MY	MD	LF MD	LR MD	RF MD	RR MD	FY	PY	CI	MS
MF	1	0.89	0.80	0.74	0.82	0.77	0.83	0.81	0.80	0.85	0.84	−0.12	−0.07	0.86	−0.39
MY		1	0.92	0.89	0.92	0.91	0.77	0.75	0.74	0.79	0.78	−0.19	−0.11	0.87	−0.17
LF MY			1	0.75	0.89	0.78	0.67	0.68	0.62	0.69	0.68	−0.11	−0.06	0.78	−0.10
LR MY				1	0.77	0.85	0.64	0.61	0.64	0.67	0.63	−0.17	−0.12	0.77	−0.14
RF MY					1	0.77	0.68	0.69	0.64	0.75	0.68	−0.13	−0.04	0.79	−0.12
RR MY						1	0.67	0.60	0.64	0.66	0.69	−0.20	−0.19	0.79	−0.11
MD							1	0.88	0.97	0.90	0.95	−0.05	0.03	0.76	−0.65
LF MD								1	0.82	0.86	0.85	−0.01	0.02	0.75	−0.53
LR MD									1	0.86	0.91	−0.07	0.03	0.73	−0.62
RF MD										1	0.89	−0.08	−0.01	0.78	−0.53
RR MD											1	−0.07	0.03	0.77	−0.60
FY												1	0.43	−0.09	−0.11
PY													1	−0.08	−0.20
CI														1	−0.29
MS															1

Correlation coefficients marked with red color were not statistically significant. MF = milking frequency, MD = milking duration, MY = milk yield, LF = left front teat, LR = left rear teat, RF = right front teat, RR = right rear teat, FY = fat yield, PY = protein yield, CI = concentrates intake, MS = milking speed.
